# Probiotic-derived ferrichrome inhibits colon cancer progression via JNK-mediated apoptosis

**DOI:** 10.1038/ncomms12365

**Published:** 2016-08-10

**Authors:** Hiroaki Konishi, Mikihiro Fujiya, Hiroki Tanaka, Nobuhiro Ueno, Kentaro Moriichi, Junpei Sasajima, Katsuya Ikuta, Hiroaki Akutsu, Hiroki Tanabe, Yutaka Kohgo

**Affiliations:** 1 Division of Gastroenterology and Hematology/Oncology, Department of Medicine, Asahikawa Medical University, Asahikawa 078-8510, Japan; 2 Center for Advanced Research and Education, Asahikawa Medical University, Asahikawa 078-8510, Japan; 3 Department of Gastroenterology, International University of Health and Welfare Hospital, Nasushiobara 329-2763, Japan

## Abstract

Previous reports have suggested that some probiotics inhibit tumorigenesis and cancer progression. However, the molecules involved have not yet been identified. Here, we show that the culture supernatant of *Lactobacillus casei* ATCC334 has a strong tumour-suppressive effect on colon cancer cells. Using mass spectrometry, we identify ferrichrome as a tumour-suppressive molecule produced by *L. casei* ATCC334. The tumour-suppressive effect of ferrichrome is greater than that of cisplatin and 5-fluorouracil, and ferrichrome has less of an effect on non-cancerous intestinal cells than either of those agents. A transcriptome analysis reveals that ferrichrome treatment induces apoptosis, which is mediated by the activation of c-jun N-terminal kinase (JNK). Western blotting indicates that the induction of apoptosis by ferrichrome is reduced by the inhibition of the JNK signalling pathway. This we demonstrate that probiotic-derived ferrichrome exerts a tumour-suppressive effect via the JNK signalling pathway.

## Abstract (web summary)

Probiotics have tumour-suppressive effects in cancer cell lines and in animal models. In this study, the authors demonstrate that ferrichrome produced by *Lactobacillus casei* ATCC334 can suppress colon cancer growth inducing apoptosis via the JNK pathway.

## Graphical abstract

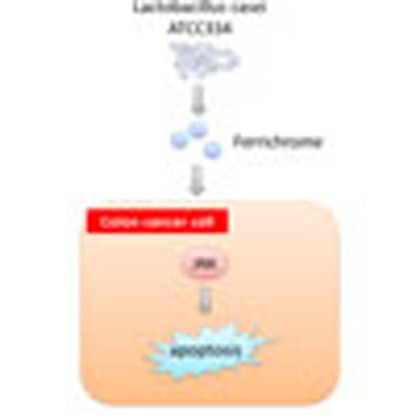

Probiotics are associated with various health benefits, including the conditioning of the intestinal microflora, suppression of excess allergic responses and tumour-suppressive effects^1^,^2^,^3^. Abnormal changes have been reported in the intestinal microflora of colon cancer patients^4^,^5^, indicating that the perturbation of the intestinal microflora is closely correlated with the initiation and progression of colon cancer cells. Moreover, probiotics, including the *Lactobacillus* and *Bifidobacterium* species, have been shown to have tumour-suppressive effects in colon cancer cell lines and in mouse/rat tumour models^6^,^7^. Thus, it is suggested that the administration of sufficient amounts of probiotics may have preventive effects against tumour initiation and progression and that it may offer therapeutic benefits. However, the tumour-suppressive effect of live probiotics is affected by numerous factors, including the conditions of the bacterial culture^8^ and differences in the populations of intestinal microbes in the host^9^,^10^.

Previous investigations concerning host–microbial interactions have shown that some of the effector molecules secreted from beneficial bacteria activate the cell survival pathways. We revealed that competence and sporulation factor (CSF) derived from *Bacillus subtilis* induces the expression of the heat shock proteins (Hsps) and activates the protein kinase B (Akt) cell survival pathway through organic cation transporter 2 (OCTN2) to exert a cytoprotective effect^11^. We subsequently found that inorganic polyphosphate isolated from the conditioned media of *Lactobacillus brevis* also induces the expression of Hsps and exerts a cytoprotective effect through the integrin β1-p38MAPK signal transduction pathway^12^. Furthermore, Yan *et al.*^13^ identified two peptides, p75 and p40, as active components that possess anti-apoptotic properties and which activate cell survival, and found that the Akt pathway is induced by *Lactobacillus GG*. These studies indicate that host–microbe interaction brings health benefits to host mammals through the mediation of specific molecules that are derived from commensal bacteria and probiotics. However, the anti-tumorigenic molecules produced by the commensal bacteria and probiotics have not been identified.

We sought to identify the tumour-suppressive molecules from the culture supernatants of the *Lactobacillus* species and successfully identified a tumour-suppressive molecule, ferrichrome (a siderophore produced by *Lactobacillus casei* ATCC334). The tumour-suppressive effect of ferrichrome on colon cancer cells was greater than or equal to that of existing anticancer drugs. In contrast, ferrichrome showed little or no growth inhibition effect on non-cancerous intestinal cells. Furthermore, we found that ferrichrome induces apoptosis through a process that is mediated by the JNK-associated induction of DNA damage-inducible transcript 3 (DDIT3) in colon cancer cells. This is the first study to identify a probiotic-derived anti-tumour molecule.

## Results

### Cancer cell growth suppression by *L. casei* supernatant

**Figure 1:**
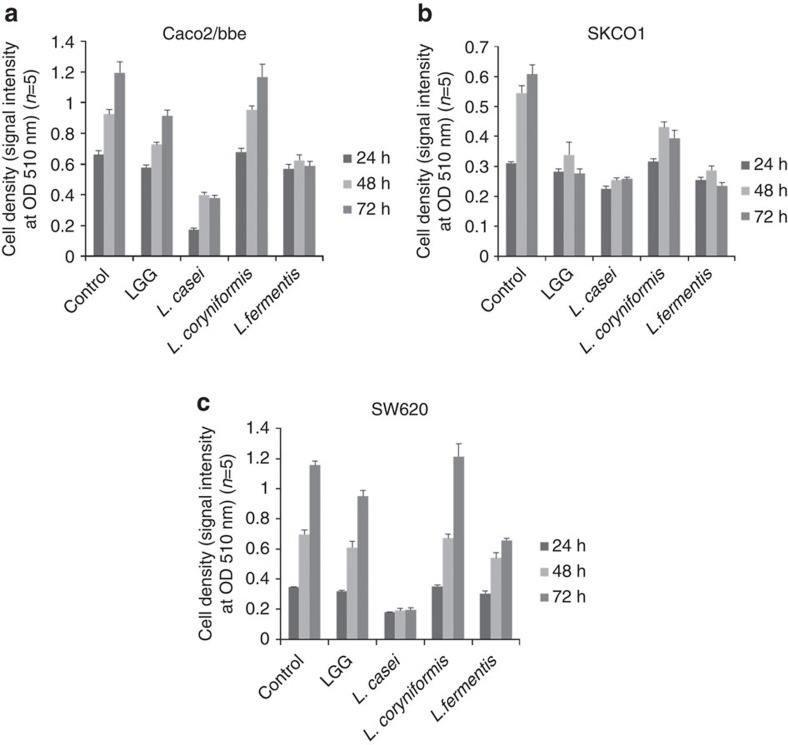
Conditioned media derived from the *Lactobacillus* spp. reduced the progression of colon cancer cells. An SRB assay revealed that the numbers of colon cancer cells, Caco2/bbe (**a**), SKCO-1 (**b**) and SW620 cells (**c**), were significantly lower in the conditioned media from the *Lactobacillus GG* ATCC53103-, *L. casei ATCC334*-, *L. coryniformis* ATCC25600- and *L. fermentis* ATCC23271-treated groups than in the control group. The strongest tumour-suppressive effect against colon cancer cells was observed in the conditioned media from the *L. casei* ATCC334 group. The error bars show the s.d. (*n*=5).

Colon cancer cells, including Caco2/bbe, SKCO-1 and SW620 cells, were incubated with the culture supernatants of *Lactobacillus GG* ATCC53103, *L. casei* ATCC334, *Lactobacillus coryniformis* ATCC25600 and *Lactobacillus fermentis* ATCC23271 to clarify their tumour-suppressive effects. All bacterial bodies and debris in the culture supernatants were removed by centrifugation and filtration using a 0.22-μm membrane. A sulforhodamine B (SRB) assay indicated that the culture supernatant of *L. coryniformis* ATCC25600 suppressed the cell growth of SKCO-1 cells, but not Caco2/bbe and SW620 cells. The bacterial culture supernatants of *Lactobacillus GG* ATCC53103, *L. casei* ATCC334 and *L. fermentis* ATCC23271 (especially the *L. casei* ATCC334 culture supernatant), suppressed the cell growth of Caco2/bbe, SKCO-1 and SW620 cells (Fig. 1a–c). These data indicate that the secreted molecule, but not the bacterial body and debris, exhibited the growth inhibition effect.

### The isolation of the tumour-suppressive fraction

**Figure 2:**
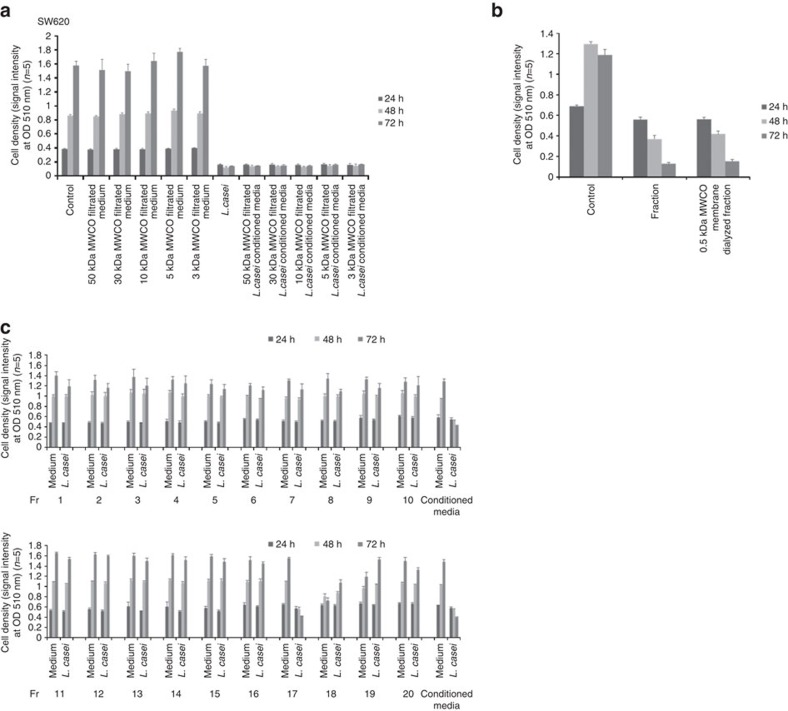
The tumour-suppressive effect of low molecular weight fraction from the *L. casei* ATCC334 culture supernatant. The tumour-suppressive effects of the *L. casei ATCC334* culture supernatant fractions were examined after filtration through 3-, 5-, 10-, 30- or 50-kDa membranes (**a**). The tumour-suppressive fraction was dialyzed using a 0.5 kDa dialysis device (**b**). A tumour-suppressive effect was confirmed in the fractions that were separated from the *L. casei ATCC334* culture supernatant by size-exclusion chromatography (**c**). The conditioned medium of *L. casei* was used as a positive control in the following study. The fractions were assessed using reverse-phase chromatography and a tumour-suppressive effect was identified in the 17th fraction by size-exclusion chromatography.

To determine the molecular weight of the tumour-suppressive molecule derived from *L. casei* ATCC334, the supernatant was separated using 50-, 30-, 10-, 5- and 3-kDa molecular weight cutoff (MWCO) membranes. An SRB assay showed that the <3 kDa fractions exhibited the growth inhibition effect (Fig. 2a). The <3 kDa fraction was further separated using a small molecule dialysis system that is capable of collecting molecules of >0.5 kDa in size. The dialyzed fraction also exhibited the growth inhibition effect (Fig. 2b). These data indicated that molecules of 0.5–3 kDa in size that were released from *L. casei* ATCC334 inhibited the growth of colon cancer cells.

**Figure 3:**
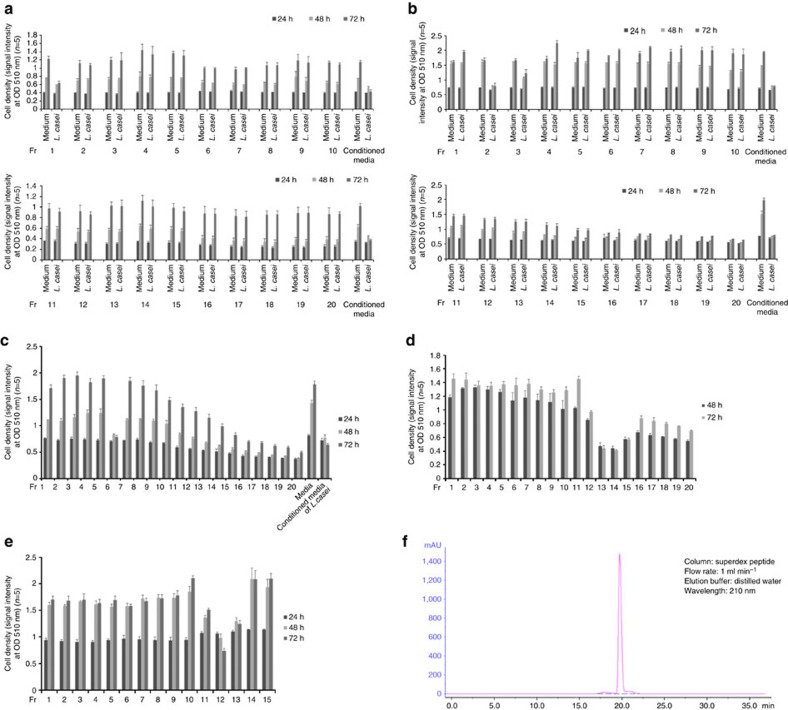
The separation of the tumour-suppressive fraction from the *L. casei* ATCC334 culture supernatant. (**a**) The fractions were assessed using DEAE anion-exchange chromatography and a tumour-suppressive effect was identified in the 1st fraction by reverse-phase chromatography (**b**). The fractions were assessed by CM cation-exchange chromatography and a tumour-suppressive effect was identified in the 2nd fraction by DEAE anion-exchange chromatography (**c**). The fractions were assessed by SP cation-exchange chromatography and a tumour-suppressive effect was identified from the 6th fraction by CM cation-exchange chromatography (**d**). The fractions were assessed by ZIC-HILIC chromatography and a tumour-suppressive effect was identified in the 13th fraction by SP cation-exchange chromatography (**e**). An HPLC chromatogram of the tumour-suppressive fraction. The sample was separated on a Superdex peptide column, eluted with distilled water at a flow rate of 1 ml min^−1^. The eluent was monitored by ultraviolet spectrophotometry at 210 nm (**f**). The error bars show the s.d. (*n*=5).

The culture supernatant of *L. casei* ATCC334 was separated using an AKTA-HPLC system and an SRB assay was performed to evaluate the tumour-suppressive effect in SW620 cells. The 17th fraction obtained by gel filtration chromatography using a Superdex peptide column significantly suppressed the growth of SW620 cells in comparison to control cells (Fig. 2c). The 17th fraction was subsequently separated using a reverse-phase column and the 1st fraction was found to have a tumour-suppressive effect (Fig. 3a), indicating that a hydrophilic molecule exhibited the tumour-suppressive effect.

Ion exchange chromatography was performed with three columns, including DEAE, CM and SP columns, to further separate the 1st fraction of reverse-phase chromatography. The 2nd (separated by DEAE) (Fig. 3b), 6th (separated by CM) (Fig. 3c), 13th and 14th fractions (separated by SP) (Fig. 3d) inhibited the growth of SW620 cells, indicating that a cationic residue was contained in the structure of the tumour-suppressive molecule.

To further separate the 13th and 14th fractions of SP ion exchange chromatography, normal-phase chromatography was performed using a ZIC-HILIC column. The 12th fraction had the most marked effect (Fig. 3e). Finally, the 12th fraction of ZIC-HILIC chromatography was confirmed to have an almost purified peak by HPLC, indicating that the purified tumour-suppressive molecule derived from *L. casei* ATCC334 was contained in the fraction (Fig. 3f).

### The characterization of the molecules of the fraction

To determine the characteristics of the tumour-suppressive molecule in the isolated fraction, the fraction was digested by protease K and the effects on the growth of SW620 cells were assessed by an SRB assay. The SRB assay showed that cell growth was reduced in both the digested and non-digested fractions (Supplementary Fig. 1A), indicating that the tumour-suppressive molecule in the isolated fraction was not a protein. To elucidate whether the tumour-suppressive molecule was a peptide, we performed an amino-acid analysis using an acid hydrated fraction. However, no amino acids were detected in the analysis (Supplementary Fig. 1B). To clarify whether the fraction contained a sugar chain, HPLC was performed to detect the 2-aminopyridine (PA)-labelled sugar chain. No sugar chain signal was detected (Supplementary Fig. 1C). A silkworm larvae plasma (SLP) test was performed to clarify whether the tumour-suppressive molecule in the isolated fraction was a peptidoglycan (PG); however, no PGs were detected (Supplementary Fig. 1D). The metallic element contents were then investigated by atomic absorption photometry. Some metallic elements, including iron, zinc and calcium, were detected (Table 1), suggesting that the tumour-suppressive molecule in the isolated fraction was associated with a metallic element.

### The identification of the tumour-suppressive molecule

**Figure 4:**
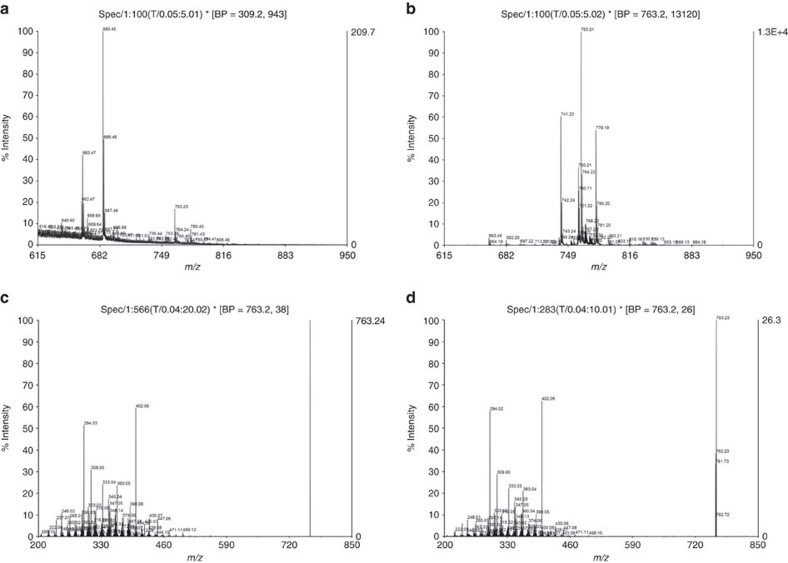
A tumour-suppressive fraction separated from the culture supernatant of *L. casei ATCC334* contained ferrichrome. The mass spectrometry analyses of the tumour-suppressive fraction (**a**) and ferrichrome (**b**) are shown. An ESI-Q-TOF analysis indicated that all of the spectrums of the *m/z* ratio of the fraction (**c**) corresponded with spectrums of the *m/z* ratio of ferrichrome (**d**).

It is known that bacteria, including *L. casei*, release hydrophilic metal chelating agents known as siderophores, which are <1.5 kDa in size, from the inside of the bacterial body to the outside to capture and incorporate metallic elements^14^. Furthermore, the genomic DNA of *L. casei* ATCC334 encodes the transmembrane subunit of the periplasmic binding protein (PBP)-dependent ATP-binding cassette (ABC) transporters, which is involved in siderophore transport (http://www.ncbi.nlm.nih.gov/gene/4421002). Two hundred sixty-two siderophores have been identified from microorganisms (SiderophoreBase, http://bertrandsamuel.free.fr/siderophore_base/index.php). The HPLC spectrum indicated that the molecule contained in the tumour-suppressive fraction was not an aromatic compound because this fraction was not detected by absorbance at 280 nm. Taken together, the tumour-suppressive molecule was thought to be a strongly hydrophilic siderophore, of 0.5–3 kDa in molecular size, because the molecule was captured by ZIC-HILIC chromatography, but not by reverse-phase chromatography. An electrospray ionization (ESI)-time of flight (TOF) analysis indicated an *m/z* ratio of 763.2 (Fig. 4a). Because a siderophore, ferrichrome, has a molecular weight at 740, the *m/z* ratio corresponds to ferrichrome monosodium salt ([M+Na]^+^ of ferrichrome) (Fig. 4b), which is not an aromatic molecule and which does not contain hydrophobic residues. An ESI- Quadrupole (Q)-TOF analysis indicated that the entire spectrum of the *m/z* ratio of the fraction (Fig. 4c) corresponded to the spectrum of the *m/z* ratio of [M+Na]^+^ of ferrichrome (Fig. 4d).

### The inhibition of colon cancer progression by ferrichrome

**Figure 5:**
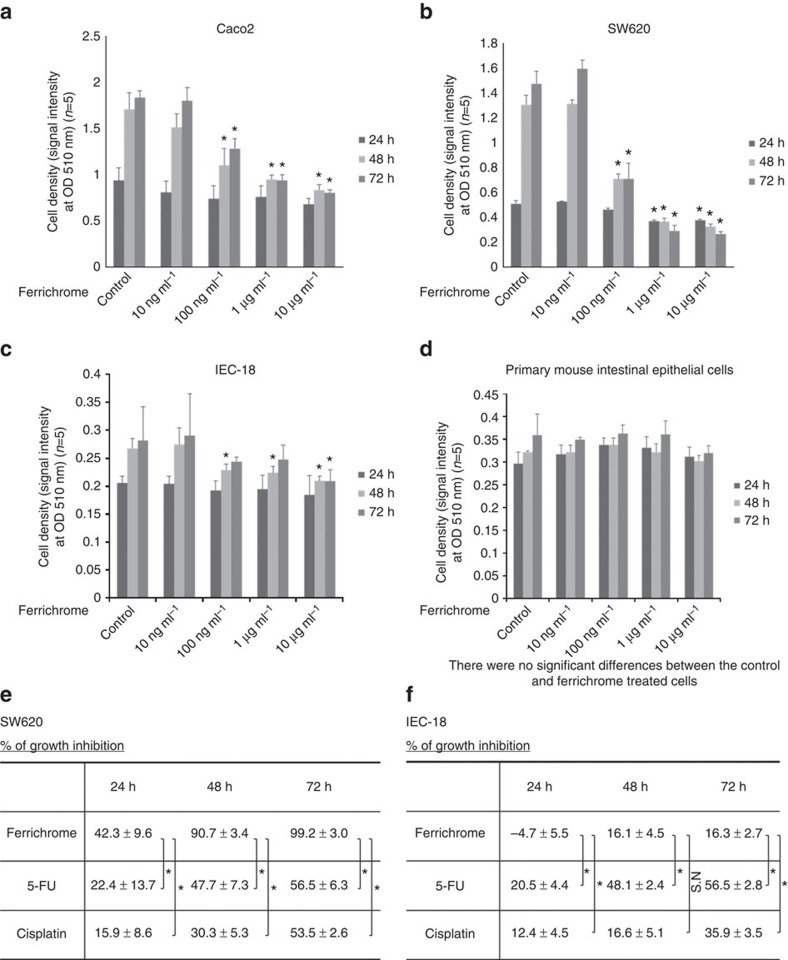
Ferrichrome exhibited tumour-suppressive effects in colon cancer cells, but not in normal epithelial cells. Ferrichrome reduced cellular progression in a dose-dependent manner in Caco2/bbe (**a**) and SW620 cells (**b**). Ferrichrome did not affect the cell growth of IEC-18 cells (**c**) or the primary cultures of intestinal cells (**d**). 5-FU and cisplatin reduced the cell growth in SW620 (**e**) and IEC-18 cells (**f**). *P<0.05 by Student's *t*-test. The error bars show the s.d. (*n*=5).

An SRB assay indicated that, at concentrations of >100 ng ml^−1^, ferrichrome reduced the cellular proliferation of Caco2 and SW620 cells (Fig. 5a,b). To determine whether the anti-tumour effect of *L. casei* was mediated by the secretion of ferrichrome, ferrichrome was precipitated with binding protein lipocalin-2 (LCN2) or ferrichrome permease (ARN1). An SRB assay revealed that the tumour-suppressive effect of the culture supernatant was reduced by treatment with these binding proteins, indicating that ferrichrome mediates the tumour-suppressive effect of *L. casei* ATCC334 (Supplementary Table 1). To assess its toxicity, ferrichrome was added to non-cancer cells. Ferrichrome was incubated with the IEC-18 cell line and with primary cultured cells derived from the mouse small intestine. The cell growth reduction effect of ferrichrome was observed at concentrations of >1 μg ml^−1^ in IEC-18 cells (Fig. 5c), but no significant effects were observed in the growth of primary cultured cells (Fig. 5d). The effects of ferrichrome on the growth of SW620 and IEC-18 cells were compared with those of anticancer drugs, including 5-fluorouracil (5-FU) and cisplatin. An SRB assay revealed that the tumour-suppressive effect of ferrichrome was greater than that of anticancer drugs in SW620 cells, and that ferrichrome had less effect on IEC-18 cells (Fig. 5e,f). These results suggest that ferrichrome suppressed the growth of colon cancer cells, while exhibiting less harmful effects in normal intestinal epithelial cells.

### Tumour growth suppression by ferrichrome *in vivo*

**Figure 6:**
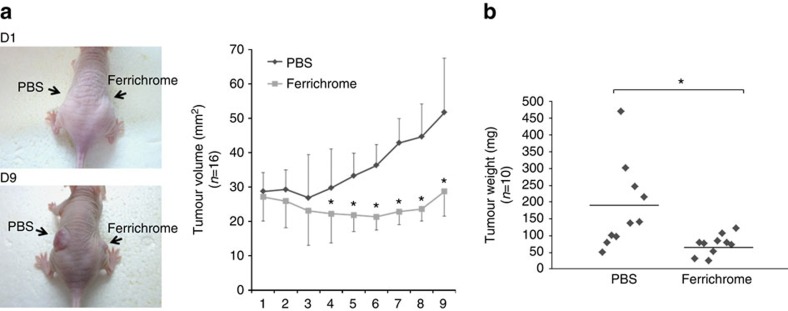
Ferrichrome inhibited tumour progression in a mouse xenograft model. In the xenograft model, the enlargement of the tumours in the ferrichrome (10 μg) group was almost completely suppressed, while the tumours in the control group were enlarged (**a**). The PBS-treated tumours were heavier than the ferrichrome-treated-tumours (**b**). *P<0.05 by Student's *t*-test. The error bars show the s.d. (**a**, *n*=16; **b**, *n*=10).

A suspension of 2 × 10^6^ of SW620 cells was injected into the backs of nude mice to build a xenograft model to confirm the suppressive effects of ferrichrome on colon cancer progression *in vivo*. Ferrichrome or PBS was injected into the tumours and the tumour sizes were measured each day. The tumour growth of the ferrichrome-treated group was significantly suppressed in comparison to the PBS-injected group (Fig. 6a,b).

### Activation of the ER stress response pathway by ferrichrome

**Figure 7:**
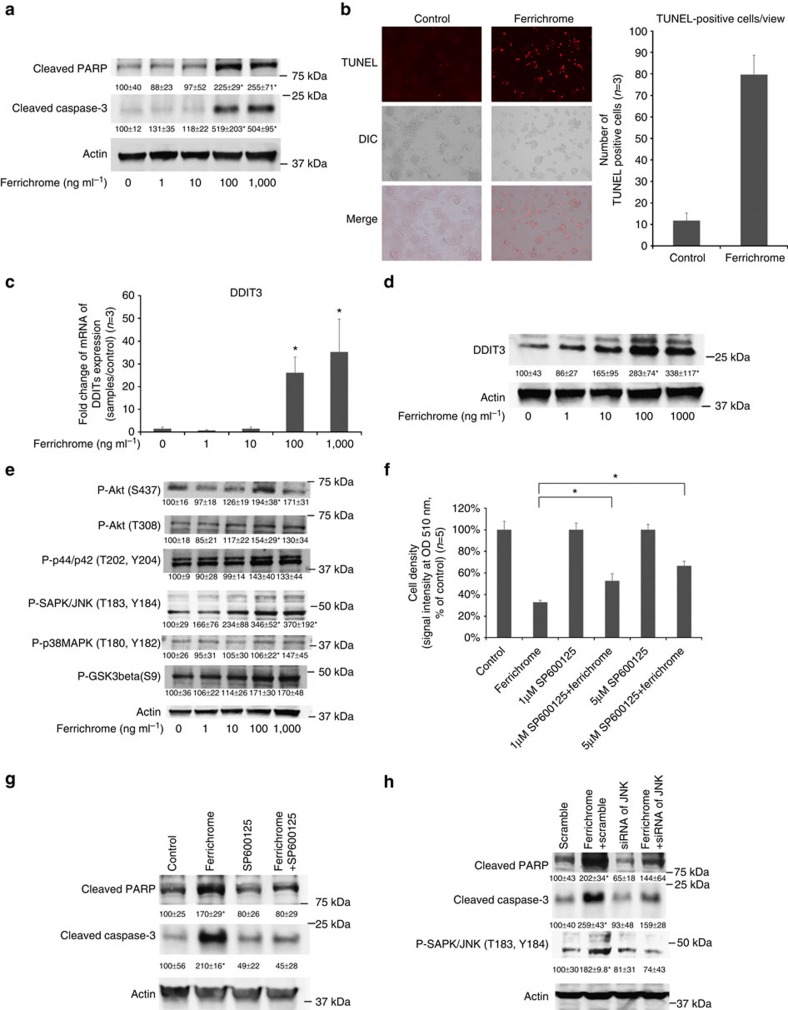
Ferrichrome-induced apoptosis is mediated by the ER stress-responsive-JNK pathway. The expression levels of cleaved caspase-3 and PARP in SW620 cells were increased by ferrichrome treatment in a dose-dependent manner (**a**). TUNEL-positive cells in SW620 cells were increased by ferrichrome treatment (0.1 μg ml^−1^). The photographs were taken under a high-power view (× 200) (**b**). DDIT3 expression was assessed using a quantitative RT–PCR (**c**) and western blotting (**d**). DDIT3 was found to be highly induced (in a dose-dependent manner) in ferrichrome-treated SW620 cells. A western blotting analysis revealed the activation of the JNK signal transduction pathway in ferrichrome-treated (0.1 μg ml^−1^) SW620 cells (**e**). The tumour-suppressive effect was reduced by the inhibition of JNK activation (**f**). A Western blotting analysis showed the elimination of cleaved caspase-3 and the induction of PARP in ferrichrome-treated (0.1 μg ml^−1^) SW620 cells by the treatment of SP600125 (**g**) or siRNA of JNK (**h**). *P<0.05 by Student's *t*-test. The error bars show the s.d. (**a**–**e** and **g**, *n*=3; **f**, *n*=5). The original unprocessed scans of the Western blots are shown in Supplementary Fig. 2.

To clarify the effects of ferrichrome on cancer cell apoptosis, the expression of cleaved caspase-3 and nuclear poly (ADP-ribose) polymerase (PARP) was assessed. A western blotting analysis revealed that the expressions of cleaved caspase-3 and PARP in the ferrichrome-treated SW620 cells were significantly increased, in a dose-dependent manner, (Fig. 7a). In addition, TUNEL staining revealed that the number of apoptotic cells in the ferrichrome-treated cells was higher than that in the control cells (Fig. 7b). These data indicated that ferrichrome exhibited its tumour-suppressive effect through the induction of apoptosis in colon cancer cells.

A high-throughput sequencing analysis was performed to determine the changes in mRNA expression that were caused by the treatment of SW620 cells with ferrichrome. Two hundred sixty-five mRNAs exhibited changes that were statistically significant and >2-fold in comparison to the control cells (Table 2; Supplementary Table 2). A pathway analysis, which was performed using the MetaCore software programme, indicated that the endoplasmic reticulum stress (ER) response pathway was involved in these mRNA changes in the ferrichrome-treated cells. The mRNA of DNA damage-inducible transcript 3 (DDIT3), which is known to be a regulator of endoplasmic reticulum stress-mediated apoptosis^15^, was the most upregulated of these mRNAs (Tables 3 and 4, Supplementary Tables 3,4). An RT–PCR and a western blotting analysis confirmed the dose-dependent upregulation of the DDIT3 mRNA and protein levels, respectively, in ferrichrome-treated cells (Fig. 7c,d). To identify the signal transduction pathway that is associated with the tumour-suppressive effect of ferrichrome, the changes of the p44/42 MAPK (ERK), protein kinase B (Akt), c-jun N-terminal kinase (JNK), p38MAPK and Glycogen synthase kinase 3β (GSK3β) signalling pathways were assessed. A western blotting analysis showed that pJNK was upregulated, in a dose-dependent manner, in ferrichrome-treated SW620 cells (Fig. 7e). An SRB assay indicated that treatment with SP600125, a JNK pathway inhibitor, reduced the tumour-suppressive effect of ferrichrome (Fig. 7f). A western blotting analysis showed that ferrichrome mediated the induction of cleaved caspase-3 and that PARP expression was reduced by treatment with SP600125 and the siRNA of JNK (Fig. 7g,h). These data indicate that ferrichrome treatment induces apoptosis in SW620 cells through the activation of the JNK-DDIT3-mediated apoptotic pathway.

## Discussion

The present study revealed that the conditioned media of *L. casei* ATCC334, which included no bacterial body components, inhibited the cell growth of colon cancer cells, suggesting that the bacteria secreted some tumour-suppressive molecules in the conditioned media. Notably, after the separation of the conditioned media using various columns, a tumour-suppressive molecule, ferrichrome, was identified from the conditioned media of the bacteria. Ferrichrome was found to strongly induce the apoptosis of colon cancer cells. While previous studies have shown that the administration of certain probiotics inhibits the progression of cancer cells *in vitro* and in animal models^6^,^7^, no molecules have been found to mediate the anti-tumour effects of such probiotics. This is the first study to identify a tumour-suppressive molecule from the conditioned media of probiotics. The present study also demonstrated that ferrichrome did not affect the cell growth of IEC-18 cells derived from the normal rat small intestine or primary cultured cells derived from the normal mouse small intestine, suggesting that the molecule has a lesser effect on non-cancerous cells. An anti-tumour agent may therefore be developed using ferrichrome.

Our SRB assay revealed that the tumour-suppressive effect of ferrichrome in colon cancer cells was greater than or equal to that of 5-FU and cisplatin (Fig. 5e,f). Our xenograft study revealed that the injection of ferrichrome strongly inhibited solid tumour development *in vivo* (Fig. 6). These data indicate that ferrichrome is an attractive anticancer drug candidate. However, these data were obtained with the direct treatment of cancer cells. The stability and delivery of ferrichrome *in vivo* remain major problems in relation to its use in the clinical setting. Thus it is necessary to determine a suitable method of drug delivery and to clarify the metabolism of ferrichrome *in vivo* in order for it to be used in the development of anticancer drugs and effective cancer therapeutics.

Our transcriptome analysis showed that ferrichrome treatment altered the expression of 265 mRNAs in SW620 cells. Furthermore, a pathway analysis using the MetaCore software programme revealed that the ER stress response pathway, which was activated by ferrichrome treatment, was probably involved in the apoptosis of SW620 cells. Among the molecules associated with the ER stress response pathway, the expression of DDIT3 mRNA showed the highest change in response to ferrichrome treatment (Table 3, Supplementary Table 3). This finding was confirmed by a qRT–PCR and western blotting (Fig. 7c,d). DDIT3 is known to induce apoptosis through Bax–Bak mitochondrial permeabilization and JNK signalling^15^. Our western blotting analysis of the signal transduction-related molecules also revealed JNK activation in ferrichrome-treated SW620 cells (Fig. 7e). Furthermore, the level of ferrichrome-mediated apoptosis was reduced by the inhibition of the JNK pathway, illustrating that ferrichrome exhibits its pro-apoptotic function through the DDIT3-JNK signalling-mediated ER stress response pathway.

Our gene silencing experiment revealed that the induction of cleaved caspase-3 and PARP by ferrichrome was reduced by the inhibition of JNK (Fig. 7h). The activation of JNK was almost suppressed by the RNA silencing of JNK. The induction of cleaved caspase-3 and PARP, but not JNK silencing, was completely suppressed by SP600125 (Fig. 7g,h). These data indicate that the JNK pathway, as well as unknown signalling pathways that can be affected by SP600125, were activated by ferrichrome and induced colon cancer cell apoptosis. Further studies will be needed to clarify the other forms of signalling activation that can be affected by ferrichrome treatment.

Mass spectrometry revealed that the tumour-suppressive fraction contained ferrichrome. However, we detected the spectrum of ferrichrome, and two other molecules were additionally detected (Fig. 4a), while an HPLC spectrum analysis of the tumour-suppressive fraction indicated the purity of the tumour-suppressive molecule (Fig. 3f). In addition to ferrichrome, these impurities were ionized, indicating that the spectrum of ferrichrome may be suppressed by the inhibition of ionization. Furthermore, an SRB assay revealed that the tumour-suppressive effect of the culture supernatant was reduced by ARN1 and LCN2 treatment. However, the cell growth was not completely recovered (Supplementary Table 1). Some other molecules may collaborate with ferrichrome to suppress tumour cell growth. To clarify how ferrichrome contributes to the tumour-suppressive function of *L. casei*, it will be necessary to perform a further analysis using an *L. casei* strain with a mutated ferrichrome synthesis pathway.

The present study demonstrated that ferrichrome was the molecule responsible for the inhibition of colon cancer cell progression that was observed with *L. casei* ATCC334, suggesting the existence of a novel anti-tumour mechanism that is mediated by the release of effective molecules that are produced by probiotics. We previously identified two effective molecules that are derived from probiotics, CSF and polyphosphate, which relieve intestinal inflammation^11^,^12^,^16^,^17^,^18^,^19^. Yan *et al.*^13^ also identified two peptides that inhibit the apoptosis of the intestinal epithelia. Some effective molecules that are secreted from probiotics have been identified and are expected to be used as new drugs in the treatment of intestinal disorders, including intestinal inflammation and neoplasia.

In summary, we demonstrated that ferrichrome is the molecule that is responsible for inhibiting the progression of colon cancer cells via JNK–DDTI3-mediated apoptosis. The present study also demonstrated the safety of ferrichrome in regard to the cell growth of non-cancerous cells, including IEC-18 and primary cultured cells derived from the mouse small intestine. Additionally, we found that the anti-tumour effect of ferrichrome against colon cancer cells was greater than or equal to that of anticancer drugs, including 5-FU and cisplatin. Thus, ferrichrome might be a practical anti-tumour agent that can be used to inhibit the progression of colon cancer.

## Methods

### Cell culture

All of the cell lines were purchased from ATCC. Human colon cancer cell lines, Caco2_bbe_, SKCO-1 and SW620 were grown in high-glucose Dulbecco's Modified Eagle's Medium (DMEM) (Caco2_bbe_, SKCO-1) or Roswell Park Memorial Institute (RPMI) 1640 (SW620) supplemented with 10% (vol/vol) fetal bovine serum (FBS), 2 mM L-glutamine, 50 U ml^−1^ penicillin and 50 μg ml^−1^ streptomycin in a humidified atmosphere containing 5% CO_2_. IEC-18 cells (a rat intestinal epithelial cell line) were grown in DMEM supplemented with 5% (vol/vol) fetal bovine serum (FBS), 1 U insulin, 2 mM L-glutamine, 50 U ml^−1^ penicillin and 50 μg ml^−1^ streptomycin. Primary cultured cells were constructed by methods that have been described previously^20^. The cells were obtained from the mucosal layer of the small intestine in the mice. The cells were plated on 6- or 12-well plates at a density of 10^5^ cells cm^−2^.

### Microorganisms

*L. GG*, *L. casei, L. coryniformis* and *L. fermentis* were purchased from the American Tissue Culture Collection (ATCC). These lactobacilli were cultured in Man–Rogosa–Sharpe (MRS) broth (Difco Laboratories, Detroit, MN) for one day at 37 °C. Each of the bacteria was then cultured in MEM for another day.

### The isolation of the tumour-suppressive molecule

The culture medium was centrifuged at 5,000*g* for 10 min to obtain the culture supernatant, which was then filtered through a 0.2-μm membrane. The culture supernatants were separated with a molecular weight cutoff spin column (GE Healthcare). The >0.5 kDa fraction was obtained by dialysis with a Micro Float-A-Lyzer Dialysis Device (Spectrum Laboratories). The culture supernatant was separated using an AKTA Design HPLC system (GE Healthcare) using a Superdex peptide column (GE Healthcare) and eluted with distilled water at a flow rate of 1 ml min^−1^. The fraction was applied to an L-column (Chemicals Evaluation and Research Institute, Japan) and eluted with 0.1% formic acid and 0.1% formic acid/acetonitrile in a linear gradient at a flow rate of 0.2 ml min^−1^. Ion exchange chromatography was performed using a HiTrap-DEAE (GE Healthcare), CM (GE Healthcare) and an SP column (GE Healthcare). The elution buffer for DEAE was 0 M and 1.0 M NaCl in 20 mM Tris-HCl (pH 8.0). The elution buffer for SP and CM was 0 M and 1.0 M NaCl in 50 mM acetic acid in a linear gradient at a flow rate of 1 ml min^−1^. Finally, the sample was separated using a ZIC-HILIC column (Merck Millipore, Germany) and eluted with 70% acetonitrile and 10% acetonitrile in a linear gradient at a flow rate of 0.5 ml min^−1^. The eluent was monitored by ultraviolet spectrophotometry at 210 nm.

### Sugar chain analysis

A PA-labelled sugar chain was generated using a BlotGlyco kit (Sumitomo Bakelite Co., Ltd) according to the manufacturer's instructions. The culture supernatant was separated using a Superdex peptide column and eluted with distilled water at a flow rate of 1 ml min^−1^. The eluent was monitored by ultraviolet spectrophotometry at 400 nm.

### SRB assay

At 24 h before stimulation, the cells were seeded on 96-well microplates at 1.0 × 10^4^ cells per well. The cells were fixed in 5% trichloroacetic acid (TCA) for 1 h at 4 °C and washed four times in distilled water. The microplates were then dehydrated at room temperature, stained in 100 μl per well of 0.057% (wt/vol) SRB powder/distilled water, washed 4 times in 0.1% acetic acid and re-dehydrated at room temperature. The stained cells were lysed in 10 mM Tris-buffer and the optical density (OD) was measured at 510 nm.

### SLP test

The peptidoglycan concentration was assessed using an SLP reagent set (Wako Pure Chemical Industries, Ltd, Japan) according to manufacturer's instructions. Samples were mixed in SLP solution and incubated at room temperature until the colour of the melanin was visualized.

### Amino-acid analysis

Samples were mixed with norvaline (OPA) and sarcosine (FMOC), treated with 6 N HCl and hydrolyzed at 110 for 24 h. Next, the samples were mixed in 80% methanol and filtered using a 0.2 μm filter. Forty microliters of each sample was mixed with 10 μl of 100 mM Na_2_B_4_O_7_ (pH10.2) (boric acid buffer) and labelled with 4 μl of OPA-3MPA (1 ml of 10 mg ml^−1^ OPA/boric acid buffer+15 μl of 3-mercaptopropionic acid) and 4 μl of 6 mM FMOC/acetonitrile (ACN). Chromatography was performed using an Agilent Poroshell 120 EC-C18 (3.0 × 150 mm 2.7 μm). The start buffer was 20 mM Na_2_HPO_4_ (pH7.6) and the elution buffer was CH_3_OH 45%+ACN 45%+H_2_O 10% in a linear gradient at a flow rate of 0.425 ml min^−1^.

### Mass spectrometry

The fraction and ferrichrome were diluted in methanol and injected into a Nano Frontier elD Liquid Chromatography Mass Spectrometer (Hitachi High-technologies, Japan) at 3 μl min^−1^. Detection was performed by mass spectrometry as follows: the instrument mode was time of flight (TOF) and the ionization type was micro-ESI. The mass range was set from 100 to 2000 Da in the full-scan positive ion mode. The product ion spectrum of ESI-Q-TOF, as a precursor ion, was obtained with an *m/z* ratio of 763.2. The drying gas flow was 1.0 l min^−1^ and the Needle/Spray potential was 5,800 V. The AP lens settings were as follows: Ex potential, 110 V; AP1 temperature, 140 °C; AP2 potential, 48 V; and AP2 temperature, 140 °C.

### Ferrichrome

Ferrichrome was purchased from Sigma-Aldrich.

### Transcriptome analysis

The ribosomal RNA was obtained using a RiboMinus Eukaryote System v2 (Life Technologies), and the RNA libraries were generated using an Ion Total RNA-Seq Kit v2 (Life Technologies). The RNA was digested by RNase III, transcribed, and then amplified using a platinum PCR supermix high-fidelity enzyme. The maker sequences were labelled at both sides of the fragmented cDNA. The concentration of the library was adjusted to 2.5 pM. The RNA libraries were then processed for an emulsion PCR using an Ion OneTouch system and an Ion OneTouch 200 Template Kit v3 (Life Technologies). Template-positive Ion Sphere particles were enriched and purified for the sequencing reaction with an Ion OneTouch ES system (Life Technologies). The template-positive Ion Sphere Particles were then applied on Ion PI Chips (Life Technologies), and a high-throughput sequencing reaction was performed using an Ion Proton Semiconductor sequencer (Life Technologies). All of the sequencing data were mapped on a human reference genome sequence (GRCh37/hg19) using the Torrent Suite software programme (Life technologies). The expression analysis for each sample was imported into the CLC Genomics Workbench software programme (CLC bio, Aarhus, Denmark), and the significance of the differences among the samples was determined using an unpaired *t*-test. The mRNA with significantly changed expression was imputed to the MetaCore software programme, and the associated signalling pathways were identified.

### Real-time PCR

Total RNA was extracted using an RNeasy mini kit (Qiagen, Valencia, CA, USA) from control or ferrichrome-treated cells. The mRNAs were reverse transcribed using a high-capacity cDNA reverse transcription kit (Applied Biosystems, Foster City, CA, USA). The reaction was carried out at 25 °C for 10 min, 37 °C for 120 min and 85 °C for 5 s. The cDNA was amplified using the specific primer for DDIT3 (purchased from Applied Biosystems, Assay ID: Hs00358796) and signal detection was performed in triplicate using an Applied Biosystems 7300 Real-Time PCR System. The assay was performed with initial denaturation at 95 °C for 10 min, followed by 40 PCR cycles of 95 °C for 10 s and 60 °C for 1 min. The average mRNA expression was normalized to the 18S rRNA expression (Applied Biosystems).

### Western blotting

Proteins lysates were prepared from control or ferrichrome-treated cells using a mammalian cell extraction kit (BioVision, Mountain View, CA, USA). The protein concentration was determined using a Bio-Rad Protein Assay Dye Reagent Concentrate (Bio-Rad, Hercules, CA, USA). Equal amounts of protein samples were separated using SDS–PAGE (12.5%), blotted onto a nitrocellulose membrane and then blocked in PBS with 0.05% (vol/vol) Tween 20 (T-PBS) containing 1% (wt/vol) bovine serum albumin (BSA). The blots were incubated overnight at 4 °C with primary antibodies. The primary antibodies of phospho-Akt (S473; #4051, T308; #4056), JNK (#9251), ERK (#4377), p38MAPK (#9215), GSK3β (#9336), cleaved caspase-3 (#9661) and PARP (#9452) were purchased from Cell Signaling Technology. All of the antibodies were diluted at 1/1,000 in PBS with 0.05% T-PBS containing 1% (wt/vol) BSA and incubated with blots overnight at 4 °C followed by incubation with horseradish peroxidase (HRP) conjugated secondary antibodies (R&D systems, Minneapolis, MN, USA) for 1 h at room temperature. Each membrane was washed in T-PBS, and then developed using the Super-Signal West Pico enhanced chemiluminescence system (Thermo Science). The chemiluminescence signal was detected using a Luminescent Image Analyzer LAS-3,000 imaging system. The averaged protein expression was normalized to the actin expression (BD Transduction Laboratories, Lexington, KY, USA).

### Xenografts

The protocols of the animal experiments were approved by the Asahikawa Medical University Institutional Animal Care and Use Committee. SW620 cells (2 × 10^6^ cells) were injected into male BALB/c nude mice. Ferrichrome (10 μg) treatments were administered daily, starting the day after the injection of SW620 cells.

### TUNEL staining

The cells were plated on chamber slides. The slides were fixed in 4% paraformaldehyde and washed extensively with PBS. The slides were stained using an *In Situ* Cell Death Detection Kit and TMR red (Roche Diagnostic, Indianapolis, IN, USA) according to manufacturer's instructions. The cells were mounted with an anti-fade mounting medium, and the TUNEL-positive cells were visualized by fluorescence microscopy (KEYENCE Corporation).

### Recombinant His-tagged ARN1

The genomic DNA of *Saccharomyces cerevisiae* was purchased from Merck Millipore Corporation and the ARN1 coding sequence was amplified by a PCR using a primer set in which the 5′ end of the upstream region contained the EcoRI restriction site and the downstream region contained the XhoI restriction site (sense, 5′-ggaattgaattcgatggagtctgttcactctcgcgatcctgttaagg-3′, anti-sense, 5′-ggaattctcgagttcccgtttaaaagtgaattttgatggaag-3′). The EcoRI/XhoI-digested PCR product was cloned into the multi-cloning site of the pET-22b vector (Novagen). The pET-22b-inserted ARN1 coding sequence was transformed to single step (KRX) competent cells (Promega) and recombinant ARN1 was induced by adding rhamnose and isopropyl β-D-1-thiogalactopyranoside (IPTG) according to manufacturer's instructions. Recombinant ARN1 was collected using a HisLink Protein Purification System (Promega).

### Recombinant His-tagged LCN2

Recombinant human His-tagged LCN2 was purchased from R & D Systems, Inc.

### The ferrichrome-deprived culture supernatant

The culture supernatant of *L. casei* ATCC334 was added the recombinant ARN1 or LCN2 and immunoprecipitated using anti-6xHis antibody (3 μg) with a Dynabeads immunoprecipitation kit (VERITAS Corporation). The precipitant was then removed and the supernatant of the immunoprecipitation was used as the ferrichrome- or siderophore-deprived culture supernatant of *L. casei* ATCC334

### Statistical analysis

The assay data were analysed using Student's *t*-test. *P* values of <0.05 were considered to indicate statistical significance.

### Data availability

The RNA sequence data obtained in the transcriptome analysis using a high-throughput sequencer have been deposited in DNA Data Bank of Japan repository under DRA004826 (Submission), PRJDB4934 (BioProject), SAMD00053873-SAMD00053878 (BioSample), DRX057473-DRX057478 (Experiment) and DRR063232-DRR063237 (Run) accession codes. All other relevant data are within the paper and its supporting information files.

## Additional information

**How to cite this article:** Konishi, H. *et al.* Probiotic-derived ferrichrome inhibits colon cancer progression via JNK-mediated apoptosis. *Nat. Commun.* 7:12365 doi: 10.1038/ncomms12365 (2016).

## Supplementary Material

Supplementary Information Supplementary Figures 1-2 and Supplementary Tables 1-4

## Figures and Tables

**Table 1:** The metallic elements contained in the fraction.

Elements (ng ml^−1^)	AV	s.d.
Ca	88.20654	6.720905
Fe	45.04926	32.48589
Zn	65.08169	2.023086
Pb	ND	
Na	ND	
Mg	ND	

ND, not determined.

The metallic elements contained in the tumour-suppressive fraction were investigated by atomic absorption spectrophotometry.

**Table 2:**
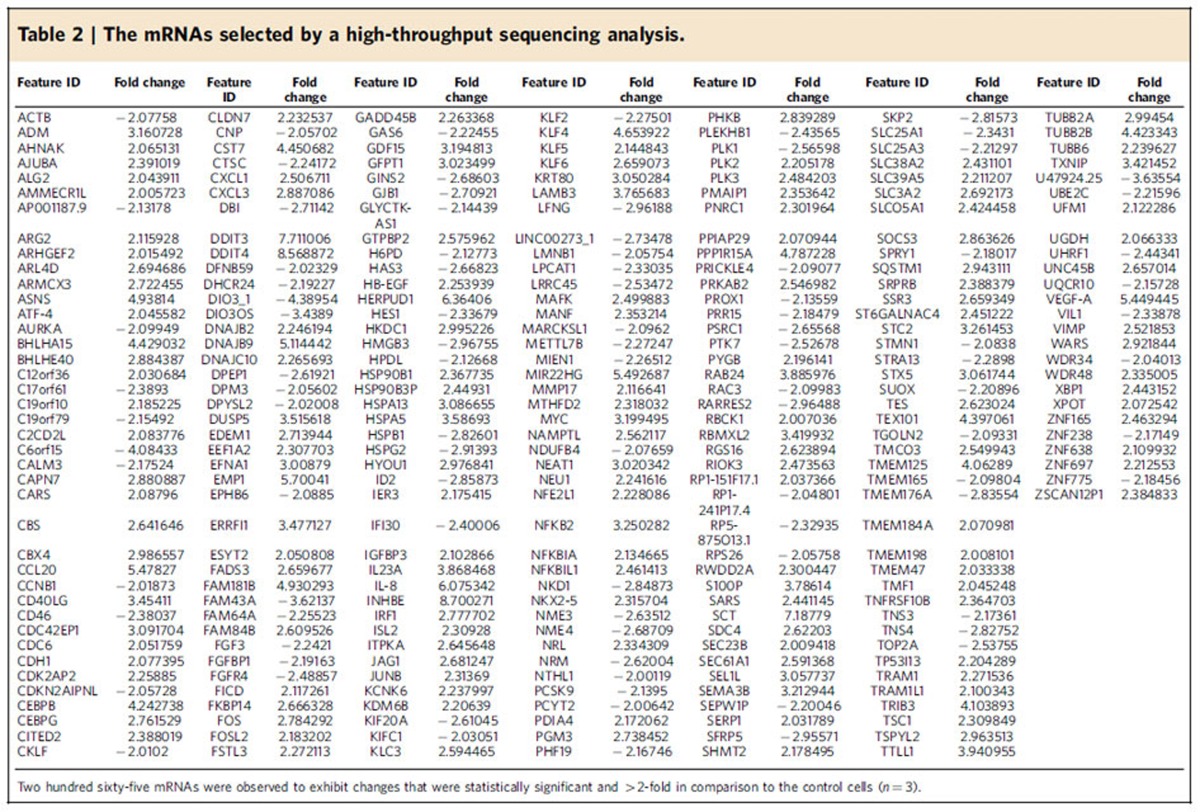
The mRNAs selected by a high-throughput sequencing analysis.

**Table 3:** The pathway analysis performed using the MetaCore software programme.

No.	Maps	*P* value	Network objects from active data
1	Apoptosis and survival_Endoplasmic reticulum stress response pathway	6.98E−08	ATF-4, Endoplasmin, GRP78, C/EBP zeta, GADD34, I-kB, EDEM, HERP, XBP1
2	Cell cycle_Role of APC in cell cycle regulation	5.12E−06	CDC18L (CDC6), CDH1, SKP2, Cyclin B, Aurora-A, PLK1
3	Apoptosis and survival_Role of PKR in stress-induced apoptosis	9.34E−06	ATF-4, IRF1, C/EBP zeta, NFKBIA, I-kB, NF-kB, c-Myc
4	Immune response_MIF-mediated glucocorticoid regulation	1.21E−05	NFKBIA, I-kB, NF-kB, IL-8, c-Fos
5	Development_Glucocorticoid receptor signalling	1.90E−05	HSP90, HSP70, NFKBIA, NF-kB, C/EBPbeta
6	Immune response_IL-17 signalling pathways	2.15E−05	CCL20, GRO-1, I-kB, NF-kB, C/EBPbeta, IL-8, c-Fos
7	IGF family signalling in colorectal cancer	2.15E−05	IBP, I-kB, NF-kB, VEGF-A, IL-8, IBP3, c-Fos
8	Apoptosis and survival_Anti-apoptotic TNFs/NF-kB/Bcl-2 pathway	2.63E−05	NF-kB2 (p100), Sequestosome 1(p62), NF-kB2 (p52), I-kB, NF-kB, CD40L(TNFSF5)
9	FGF signalling in pancreatic cancer	4.49E−05	NFKBIA, E-cadherin, NF-kB, HBP17, VEGF-A, c-Fos
10	p53 Signalling in Prostate Cancer	9.57E−05	DR4(TNFRSF10A), Stathmin, NOXA, DR5(TNFRSF10B), IBP3
11	LRRK2 in neurons in Parkinson's disease	9.57E−05	HSP90, eEF1A2, ACTB, eEF1A, Actin cytoskeletal
12	Cell cycle_ESR1 regulation of G1/S transition	9.57E−05	SKP2, NCOA3 (pCIP/SRC3), c-Myc, Skp2/TrCP/FBXW, c-Fos
13	Immune response_Lipoxins and Resolvin E1 inhibitory action on neutrophil functions	1.28E−04	NFKBIA, I-kB, NF-kB, IL-8, c-Fos
14	Immune response_Role of PKR in stress-induced antiviral cell response	1.53E−04	IRF1, NFKBIA, I-kB, NF-kB, IL-8, c-Myc
15	Development_ERBB-family signalling	2.17E−04	HB-EGF, I-kB, NF-kB, c-Myc, c-Fos
16	Immune response_TSLP signalling	2.17E−04	NFKBIA, Claudin-7, NF-kB, IL-8, c-Myc
17	Immune response_Neurotensin-induced activation of IL-8 in colonocytes	3.09E−04	I-kB, NF-kB, Calmodulin, IL-8, c-Fos
18	Development_Role of IL-8 in angiogenesis	3.17E−04	HB-EGF, I-kB, NF-kB, VEGF-A, IL-8, c-Fos
19	Impaired inhibitory action of lipoxins and Resolvin E1 on neutrophil functions in CF	3.46E−04	NFKBIA, I-kB, NF-kB, IL-8, c-Fos
20	Signal transduction_AKT signalling	3.46E−04	HSP90, Hamartin, I-kB, NF-kB, c-Myc

The endoplasmic reticulum stress response pathway was markedly altered in the ferrichrome-treated cells (*n*=3).

**Table 4:** The endoplasmic reticulum (ER) stress response-related molecules with significantly altered expression.

Gene symbol	Object type	Description	Integrity biomarker	Signal (fold change)	*P* value
DDIT3	Transcription factor	DNA damage-inducible transcript 3 protein	DNA damage-inducible transcript 3	7.711006	1.05E−04
HERPUD1	Generic binding protein	Homocysteine-responsive endoplasmic reticulum-resident ubiquitin-like domain member 1 protein	Homocysteine-responsive endoplasmic reticulum-resident ubiquitin-like domain member 1 protein	6.36406	1.37E−04
PPP1R15A	Generic binding protein	Protein phosphatase 1 regulatory subunit 15A	Protein phosphatase 1 regulatory subunit 15A	4.787228	2.70E−04
HSPA5	Generic binding protein	78 kDa glucose-regulated protein	Heat shock 70 kDa protein 5 (glucose-regulated protein, 78 kDa)	3.58693	4.49E−04
EDEM1	Generic enzyme	ER degradation-enhancing alpha-mannosidase-like protein 1	ER degradation-enhancing alpha-mannosidase-like 1	2.713944	1.09E−03
XBP1	Transcription factor	X-box-binding protein 1	X-box-binding protein 1	2.443152	9.49E−05
HSP90B1	Generic binding protein	Endoplasmin	Endoplasmin	2.367735	2.89E−04
NFKBIA	Generic binding protein	–	Nuclear factor of kappa light polypeptide gene enhancer in B-cell inhibitor, alpha	2.134665	4.07E−04
ATF-4	Transcription factor	Cyclic AMP-dependent transcription factor ATF-4	Cyclic AMP-dependent transcription factor ATF-4	2.045582	7.35E−04

ER stress-responsive molecules, including DNA damage-inducible transcript 3 (DDIT3) and 78 kDa glucose-regulated protein, were significantly upregulated in the ferrichrome-treated cells (*n*=3).
