# Supplementary material for: Probiotic-derived ferrichrome inhibits colon cancer progression via JNK-mediated apoptosis
Source: Nat Commun. 2016 Aug 10;7:12365. doi: 10.1038/ncomms12365 (PMC4987524; doi:10.1038/ncomms12365)
Supplement: Supplementary Information — Supplementary Figures 1-2 and Supplementary Tables 1-4 [file ncomms12365-s1.pdf]

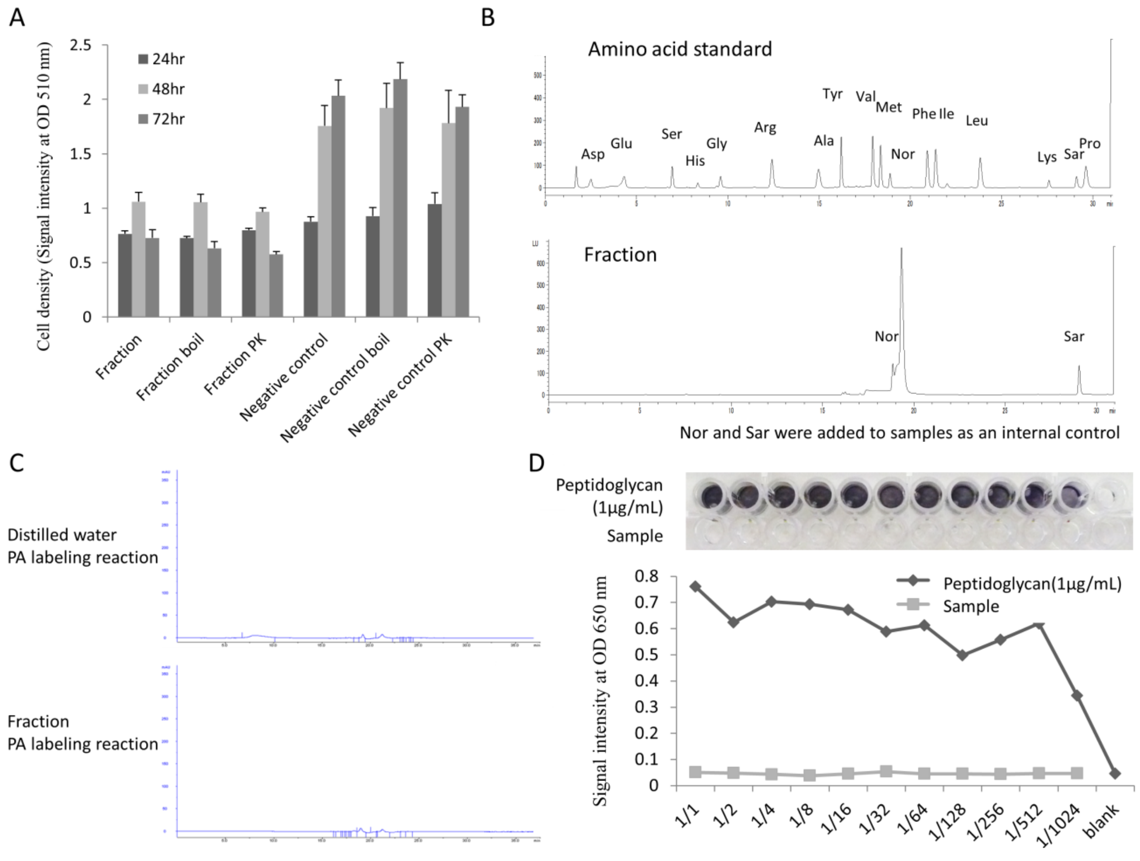

Supplementary Figure 1 A tumor-suppressive fraction separated from the culture supernatant of *L. casei* ATCC334, was not a protein, peptide glycan or a sugar chain

The tumor-suppressive fraction was heat- and protease K digestion-stable (A) (n=5). An amino acid analysis after the acid degradation of the tumor-suppressive fraction by HPLC (B). HPLC (using the PA labeling method) was performed to detect sugar chains (C). The peptide glycan content in the fraction. The peptide glycan concentration as measured by an SLP test (D).

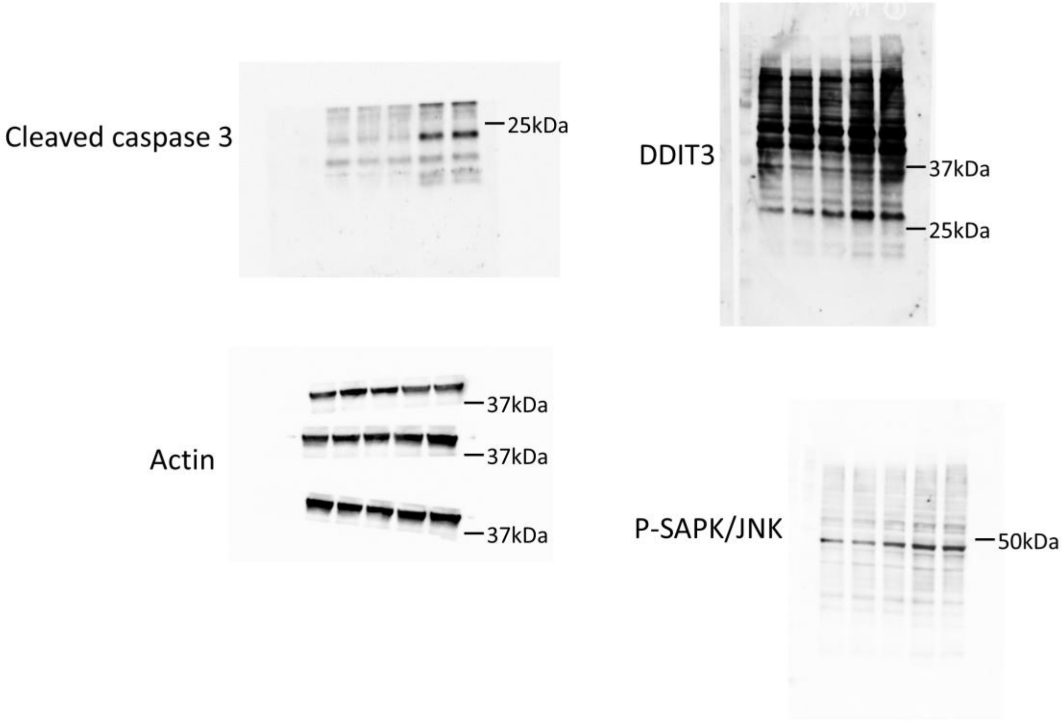

Supplementary Figure 2 Unprocessed original scans of western blots.

Supplementary Table 1 The anti-tumor effect of the ferrichrome-deprived supernatant of *L. casei* ATCC334

% of growth inhibition

	Average	Standard deviation
Culture supernatant	65.55357	8.155791
IP-ARN1	42.42957*	11.48358
IP-LCN2	38.11134*	7.30429

% of growth inhibition

	Average	Standard deviation
Ferrichrome	71.87869	8.266474
IP-ARN1	48.38782*	6.747381
IP-LCN2	40.33055*	5.574248

An SRB assay revealed that the tumor-suppressive effect of the culture supernatant of *L. casei* ATCC334 was reduced by treatment with ARN1 and LCN2 (A) (n=5). The tumor-suppressive effect of 100 ng/mL ferrichrome was reduced by treatment with ARN1 and LCN2 (B) (n=5). *$p<0.05$ by Student's *t*-test.

Supplementary Table 2 The mRNAs selected by a high-throughput sequencing analysis.

Feature ID	Fold Change	Feature ID	Fold Change	Feature ID	Fold Change	Feature ID	Fold Change	Feature ID	Fold Change	Feature ID	Fold Change	Feature ID	Fold Change
ACTB	-2.07758	CLDN7	2.232537	GADD45B	2.263368	KLF2	-2.27501	PHKB	2.839289	SKP2	-2.81573	TUBB2A	2.99454
ADM	3.160728	CNP	-2.05702	GAS6	-2.22455	KLF4	4.653922	PLEKHB1	-2.43565	SLC25A1	-2.3431	TUBB2B	4.423343
AHNAK	2.065131	CST7	4.450682	GDF15	3.194813	KLF5	2.144843	PLK1	-2.56598	SLC25A3	-2.21297	TUBB6	2.239627
AJUBA	2.391019	CTSC	-2.24172	GFPT1	3.023499	KLF6	2.659073	PLK2	2.205178	SLC38A2	2.431101	TXNIP	3.421452
ALG2	2.043911	CXCL1	2.506711	GINS2	-2.68603	KRT80	3.050284	PLK3	2.484203	SLC39A5	2.211207	U47924.25	-3.63554
AMMECR1L	2.005723	CXCL3	2.887086	GJB1	-2.70921	LAMB3	3.765683	PMAIP1	2.353642	SLC3A2	2.692173	UBE2C	-2.21596
AP001187.9	-2.13178	DBI	-2.71142	GLYCTK-AS1	-2.14439	LFNG	-2.96188	PNRC1	2.301964	SLCO5A1	2.424458	UFM1	2.122286
ARG2	2.115928	DDIT3	7.711006	GTPBP2	2.575962	LINC00273_1	-2.73478	PPIAP29	2.070944	SOCS3	2.863626	UGDH	2.066333
ARHGEF2	2.015492	DDIT4	8.568872	H6PD	-2.12773	LMNB1	-2.05754	PPP1R15A	4.787228	SPRY1	-2.18017	UHRF1	-2.44341
ARL4D	2.694686	DFNB59	-2.02329	HAS3	-2.66823	LPCAT1	-2.33035	PRICKLE4	-2.09077	SQSTM1	2.943111	UNC45B	2.657014
ARMCX3	2.722455	DHCR24	-2.19227	HBEGF	2.253939	LRRC45	-2.53472	PRKAB2	2.546982	SRPRB	2.388379	UQCR10	-2.15728
ASNS	4.93814	DIO3_1	-4.38954	HERPUD1	6.36406	MAFK	2.499883	PROX1	-2.13559	SSR3	2.659349	VEGFA	5.449445
ATF4	2.045582	DIO3OS	-3.4389	HES1	-2.33679	MANF	2.353214	PRR15	-2.18479	ST6GALNAC4	2.451222	VIL1	-2.33878
AURKA	-2.09949	DNAJB2	2.246194	HKDC1	2.995226	MARCKSL1	-2.0962	PSRC1	-2.65568	STC2	3.261453	VIMP	2.521853
BHLHA15	4.429032	DNAJB9	5.114442	HMGB3	-2.96755	METTL7B	-2.27247	PTK7	-2.52678	STMN1	-2.0838	WARS	2.921844
BHLHE40	2.884387	DNAJC10	2.265693	HPDL	-2.12668	MIEN1	-2.26512	PYGB	2.196141	STRA13	-2.2898	WDR34	-2.04013
C12orf36	2.030684	DPEP1	-2.61921	HSP90B1	2.367735	MIR22HG	5.492687	RAB24	3.885976	STX5	3.061744	WDR48	2.335005
C17orf61	-2.3893	DPM3	-2.05602	HSP90B3P	2.44931	MMP17	2.116641	RAC3	-2.09983	SUOX	-2.20896	XBP1	2.443152
C19orf10	2.185225	DPYSL2	-2.02008	HSPA13	3.086655	MTHFD2	2.318032	RARRES2	-2.96488	TES	2.623024	XPOT	2.072542
C19orf79	-2.15492	DUSP5	3.515618	HSPA5	3.58693	MYC	3.199495	RBCK1	2.007036	TEX101	4.397061	ZNF165	2.463294
C2CD2L	2.083776	EDEM1	2.713944	HSPB1	-2.82601	NAMPTL	2.562117	RBMXL2	3.419932	TGOLN2	-2.09331	ZNF238	-2.17149
C6orf15	-4.08433	EEF1A2	2.307703	HSPG2	-2.91393	NDUFB4	-2.07659	RGS16	2.623894	TMCO3	2.549943	ZNF638	2.109932
CALM3	-2.17524	EFNA1	3.00879	HYOU1	2.976841	NEAT1	3.020342	RIOK3	2.473563	TMEM125	4.06289	ZNF697	2.212553
CAPN7	2.880887	EMP1	5.70041	ID2	-2.85873	NEU1	2.241616	RP1-151F17.1	2.037366	TMEM165	-2.09804	ZNF775	-2.18456
CARS	2.08796	EPHB6	-2.0885	IER3	2.175415	NFE2L1	2.228086	RP1-241P17.4	-2.04801	TMEM176A	-2.83554	ZSCAN12P1	2.384833
CBS	2.641646	ERRFI1	3.477127	IFI30	-2.40006	NFKB2	3.250282	RP5-875O13.1	-2.32935	TMEM184A	2.070981		
CBX4	2.986557	ESYT2	2.050808	IGFBP3	2.102866	NFKBIA	2.134665	RPS26	-2.05758	TMEM198	2.008101		
CCL20	5.47827	FADS3	2.659677	IL23A	3.868468	NFKBIL1	2.461413	RWDD2A	2.300447	TMEM47	2.033338		
CCNB1	-2.01873	FAM181B	4.930293	IL8	6.075342	NKD1	-2.84873	S100P	3.78614	TMF1	2.045248		
CD40LG	3.45411	FAM43A	-3.62137	INHBE	8.700271	NKX2-5	2.315704	SARS	2.441145	TNFRSF10B	2.364703		
CD46	-2.38037	FAM64A	-2.25523	IRF1	2.777702	NME3	-2.63512	SCT	7.18779	TNS3	-2.17361		
CDC42EP1	3.091704	FAM84B	2.609526	ISL2	2.30928	NME4	-2.68709	SDC4	2.62203	TNS4	-2.82752		

CDC6	2.051759	FGF3	-2.2421	ITPKA	2.645648	NRL	2.334309	SEC23B	2.009418	TOP2A	-2.53755
CDH1	2.077395	FGFBP1	-2.19163	JAG1	2.681247	NRM	-2.62004	SEC61A1	2.591368	TP53I13	2.204289
CDK2AP2	2.25885	FGFR4	-2.48857	JUNB	2.31369	NTHL1	-2.00119	SEL1L	3.057737	TRAM1	2.271536
CDKN2AIPNL	-2.05728	FICD	2.117261	KCNK6	2.237997	PCSK9	-2.1395	SEMA3B	3.212944	TRAM1L1	2.100343
CEBPB	4.242738	FKBP14	2.666328	KDM6B	2.20639	PCYT2	-2.00642	SEPW1P	-2.20046	TRIB3	4.103893
CEBPG	2.761529	FOS	2.784292	KIF20A	-2.61045	PDIA4	2.172062	SERP1	2.031789	TSC1	2.309849
CITED2	2.388019	FOSL2	2.183202	KIFC1	-2.03051	PGM3	2.738452	SFRP5	-2.95571	TSPYL2	2.963513
CKLF	-2.0102	FSTL3	2.272113	KLC3	2.594465	PHF19	-2.16746	SHMT2	2.178495	TTLL1	3.940955

Two hundred sixty-five mRNAs were observed to exhibit changes that were statistically significant and >2-fold in comparison to the control cells (n=3).

Supplementary Table 3 The pathway analysis by the MetaCore software program.

#	Maps	p-value	Network Objects from Active Data
1	Apoptosis and survival_Endoplasmic reticulum stress response pathway	6.98E-08	ATF-4, Endoplasmin, GRP78, C/EBP zeta, GADD34, I-kB, EDEM, HERP, XBP1
2	Cell cycle_Role of APC in cell cycle regulation	5.12E-06	CDC18L (CDC6), CDH1, SKP2, Cyclin B, Aurora-A, PLK1
3	Apoptosis and survival_Role of PKR in stress-induced apoptosis	9.34E-06	ATF-4, IRF1, C/EBP zeta, NFKBIA, I-kB, NF-kB, c-Myc
4	Immune response_MIF-mediated glucocorticoid regulation	1.21E-05	NFKBIA, I-kB, NF-kB, IL-8, c-Fos
5	Development_Glucocorticoid receptor signaling	1.90E-05	HSP90, HSP70, NFKBIA, NF-kB, C/EBPbeta
6	Immune response_IL-17 signaling pathways	2.15E-05	CCL20, GRO-1, I-kB, NF-kB, C/EBPbeta, IL-8, c-Fos
7	IGF family signaling in colorectal cancer	2.15E-05	IBP, I-kB, NF-kB, VEGF-A, IL-8, IBP3, c-Fos
8	Apoptosis and survival_Anti-apoptotic TNFs/NF-kB/Bcl-2 pathway	2.63E-05	NF-kB2 (p100), Sequestosome 1(p62), NF-kB2 (p52), I-kB, NF-kB, CD40L(TNFSF5)
9	FGF signaling in pancreatic cancer	4.49E-05	NFKBIA, E-cadherin, NF-kB, HBP17, VEGF-A, c-Fos
10	p53 signaling in Prostate Cancer	9.57E-05	DR4(TNFRSF10A), Stathmin, NOXA, DR5(TNFRSF10B), IBP3
11	LRRK2 in neurons in Parkinson's disease	9.57E-05	HSP90, eEF1A2, ACTB, eEF1A, Actin cytoskeletal
12	Cell cycle_ESR1 regulation of G1/S transition	9.57E-05	SKP2, NCOA3 (pCIP/SRC3), c-Myc, Skp2/TrCP/FBXW, c-Fos
13	Immune response_Lipoxins and Resolvin E1 inhibitory action on neutrophil functions	1.28E-04	NFKBIA, I-kB, NF-kB, IL-8, c-Fos
14	Immune response_Role of PKR in stress-induced antiviral cell response	1.53E-04	IRF1, NFKBIA, I-kB, NF-kB, IL-8, c-Myc
15	Development_ERBB-family signaling	2.17E-04	HB-EGF, I-kB, NF-kB, c-Myc, c-Fos
16	Immune response_TSLP signalling	2.17E-04	NFKBIA, Claudin-7, NF-kB, IL-8, c-Myc
17	Immune response_Neurotensin-induced activation of IL-8 in colonocytes	3.09E-04	I-kB, NF-kB, Calmodulin, IL-8, c-Fos
18	Development_Role of IL-8 in angiogenesis	3.17E-04	HB-EGF, I-kB, NF-kB, VEGF-A, IL-8, c-Fos
19	Impaired inhibitory action of lipoxins and Resolvin E1 on neutrophil functions in CF	3.46E-04	NFKBIA, I-kB, NF-kB, IL-8, c-Fos
20	Signal transduction_AKT signaling	3.46E-04	HSP90, Hamartin, I-kB, NF-kB, c-Myc

The endoplasmic reticulum stress response pathway was dramatically altered in the ferrichrome-treated cells (n=3).

Supplementary Table 4 The list of endoplasmic reticulum stress markers that were significantly changed by the related molecules

Gene Symbol	Object Type	Description	Integrity Biomarker	Signal (fold change)	P-value
DDIT3	Transcription factor	DNA damage-inducible transcript 3 protein	DNA-damage-inducible transcript 3	7.711006	1.05E-04
HERPUD1	Generic binding protein	Homocysteine-responsive endoplasmic reticulum-resident ubiquitin-like domain member 1 protein	Homocysteine-responsive endoplasmic reticulum-resident ubiquitin-like domain member 1 protein	6.36406	1.37E-04
PPP1R15A	Generic binding protein	Protein phosphatase 1 regulatory subunit 15A	Protein phosphatase 1 regulatory subunit 15A	4.787228	2.70E-04
HSPA5	Generic binding protein	78 kDa glucose-regulated protein	Heat shock 70kDa protein 5 (glucose-regulated protein, 78kDa)	3.58693	4.49E-04
EDEM1	Generic enzyme	ER degradation-enhancing alpha-mannosidase-like protein 1	ER degradation-enhancing alpha-mannosidase-like 1	2.713944	1.09E-03
XBP1	Transcription factor	X-box-binding protein 1	X-box-binding protein 1	2.443152	9.49E-05
HSP90B1	Generic binding protein	Endoplasmin	Endoplasmin	2.367735	2.89E-04
NFKBIA	Generic binding protein	_	Nuclear factor of kappa light polypeptide gene enhancer in B-cells inhibitor, alpha	2.134665	4.07E-04
ATF4	Transcription factor	Cyclic AMP-dependent transcription factor ATF-4	Cyclic AMP-dependent transcription factor ATF-4	2.045582	7.35E-04

ER stress-responsive molecules, including DNA damage-inducible transcript 3 (DDIT3) and 78 kDa glucose-regulated protein, were significantly upregulated in the ferrichrome-treated cells (n=3).
